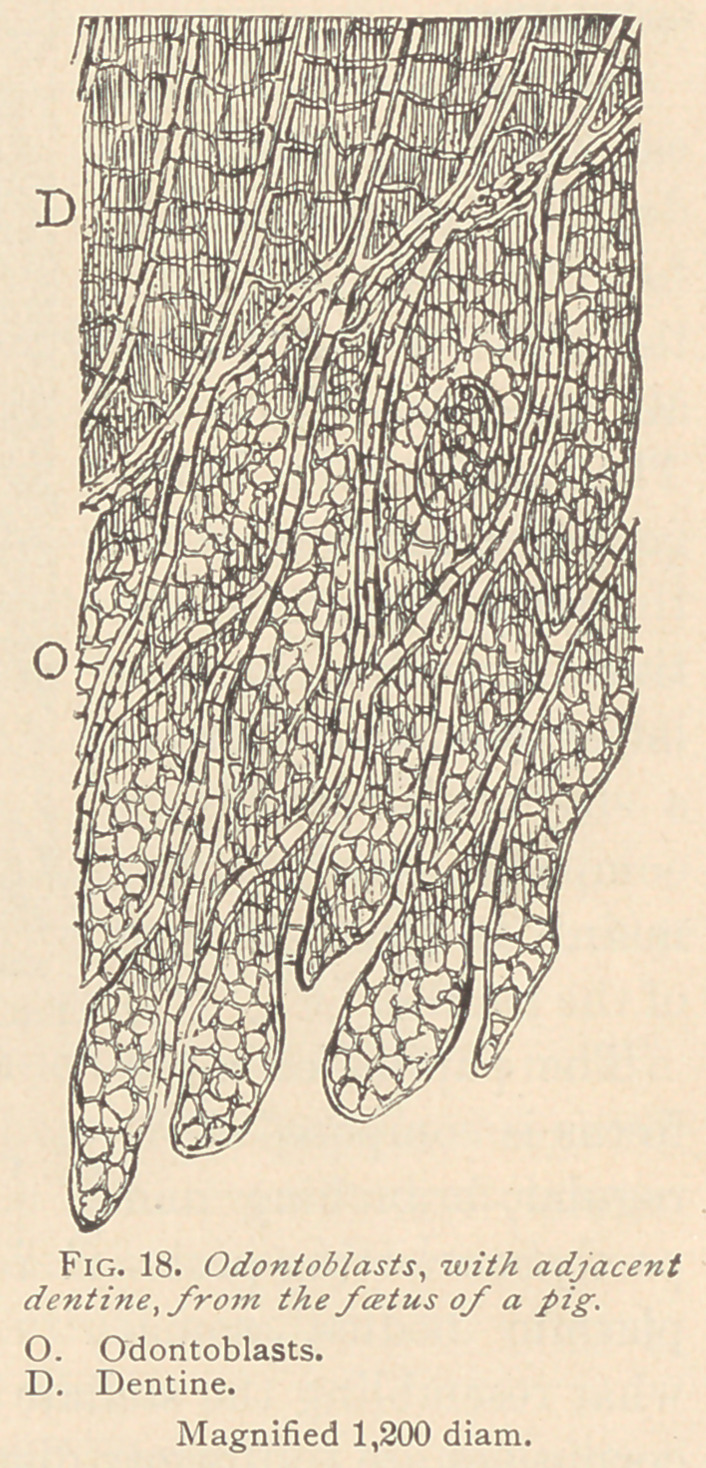# Contributions to the History of Development of the Teeth

**Published:** 1887-07

**Authors:** Carl Heitzmann, C. F. W. Bödecker


### Independent Practitioner.

Vol. VIII. July, 1887. No. 7.

Note.—No paper published or to be published in another journal will be accepted for this department. All papers must be in the hands of the Editor before the first day of the month preceding that in which they are expected to appear. Extra copies will be furnished to each contributor of an accepted original article, and reprints, in pamphlet form, may be had at the cost of the paper, press-work and binding, if ordered when the manuscript is forwarded. The Editor and Publishers are not responsible for the opinions expressed by contributors. The journal is issued promptly. on the first day of each month.

### Original Communications.

### CONTRIBUTIONS TO THE HISTORY OF DEVELOPMENT OF THE TEETH.

BY CARL HEITZMANN, M. D., AND C. F. W. BÖDECKER, D. D. S., M. D. S.

Continued From Page 290.

### II. Development of Dentine.

To prepare microscopical specimens of embryonal jaws for researches in the history of the development of the teeth, the jawbones (preferably the lower) are excised from the fœtus, care being exercised not to squeeze the specimen, and placed in a one-half of one per cent. solution of chromic acid. The liquid must be changed every third or fourth day, until the hard tissues, by the removal of the lime-salts, are sufficiently soft for cutting with a razor. All trials to determine the degree of softness of such a specimen must be made by means of a needle, and not with the fingers. The epithelial structures are especially liable to be crushed, unless handled with the utmost care. The greatest obstacle to obtaining perfect specimens is caused by the fact that the enamel organ is withdrawn from the dentinal cap, and the epithelia from the connective tissue structures, a cavity being thus formed whose walls offer no resistance to the cutting instruments, and are thus easily detached from their normal position.

After having tried different methods of imbedding, the writers came to the conclusion that the best material for this purpose is celloidine, softened in absolute alcohol, and dissolved in sulphuric ether. The specimen, previous to imbedding, must be dehydrated by immersion in absolute alcohol for twenty-four hours. After this, it is kept in a mixture of equal parts of sulphuric ether and absolute alcohol about twenty-four hours longer, when it is placed in a rather thin solution of celloidine for about two days, after which it is ready for mounting upon a cork. When such a specimen is cut, it will usually be found that the spaces between the dentine and enamel, which are due to the shrinkage of the myxomatous tissue of the enamel organ, are filled with celloidine. If, however, it is found upon cutting that such a space has not been completely filled, a very thin solution of celloidine may be poured into it. A section cutter is of great advantage, since a large number of uniform and thin specimens can be obtained in a comparatively short space of time. That made by Toma of Heidelberg, is especially to be commended. The writers would consider glycerine far superior to Canada balsam as a mounting medium, the tissues presenting a more distinct appearance. If high powers of the microscope are to be employed, only glycerine-mounted specimens will give satisfactory results.

Dentine, as is universally admitted, is strictly an offspring of connective tissue produced by the papilla, which is a formation of embryonal tissue, crowded with medullary corpuscles. (See Fig. 1, page 227.)

It begins to appear about the end of the second and beginning of the third month of intra-uterine life, at a time when the extremity of the epithelial cord has begun to flatten and assume a cup shape. The cavity of this cup is filled with the papilla (Fig. 1, P.) which sends prolongations along the outer wall of the cup, the future sack of the tooth. The more this is deepened and widened, the larger will be the papilla. If the cup of the enamel organ shows depressions corresponding to a bicuspid or molar tooth, we will find corresponding elevations upon the papilla extending into them.

The papilla is originally supplied with but few capillary blood vessels. With advancing growth, however, the vascular supply becomes greater, especially in its peripheral portions, where a delicate fibrous connective tissue is developed—the so-called tooth sack. In the seventh month of fœtal life, we observe a perfect vascular apparatus traversing the papilla, consisting of arteries, veins and capillaries. If examined with low powers of the microscope the papilla appears to be composed of small, globular, highly refracting corpuscles, between which is seen a scanty basis-substance. With high powers of the microscope, we observe globular or oblong corpuscles of small size, which are either compact and homogeneous, or possessed of a distinct reticular structure in their interior. Between small groups of such medullary corpuscles, spindle-shaped tracts appear, corresponding to the boundaries of the future territories of the myxomatous tissue, the reticulum of which in the human subject is always incomplete, never attaining the degree of development seen in the enamel organ. The largest medullary corpuscles are observed in the middle of territories surrounded by a certain amount of basis-substance. Both these corpuscles and the basis-substance have a distinct reticular structure. (Fig. 12.)

FIG. 12. *Medullary tissue of the papilla of a human embryo of four months.*

The small shining medullary corpuscles form clusters, around which we observe an irregularly developed myxomatous network. Magnified 1,200 diam.

The more the papilla increases in size and advances in development, the less frequent are the homogeneous and small medullary corpuscles, while the granular corpuscles are larger, each being surrounded by a small amount of basis-substance. Along the periphery of the papilla, however, there is invariably present a narrow zone in which the medullary corpuscles are more numerous and more shining than in the rest of the papilla. In the fifth month the outermost periphery of the papilla is frequently found to be composed of a hyaline rim, beneath which is a narrow zone of medullary corpuscles. The hyaline rim corresponds to the so-called structureless layer, or basement membrane so often seen between epithelial and connective tissue formations. When the enamel organ is detached from the papilla, and this is, as above mentioned, very frequently the case, the outer surface of the basement membrane appears beset with an extremely delicate fringe, evidently the torn connection between the papilla and the adjacent enamel organ, or the ameloblasts. High powers of the microscope reveal in the apparently structureless layer a faint reticular formation, and marks of a division into medullary corpuscles. (See Fig. 7, p. 282.)

At the beginning of the fifth month, we usually find the first traces of peculiar, elongated corpuscles along the periphery of the papilla, which are known as odontoblasts. These formations are oblong, pear, club, or spindle-shaped, arising from the coalescence of a number of medullary corpuscles, including that portion which has been previously transformed into basis-substance. The odontoblasts are sometimes seen directly beneath an already formed layer of not yet calcified dentine. The latter, in this situation, is sufficiently characterized by the presence of delicate branching dentinal canaliculi, holding the slender dentinal (Tomes) fibers. We often observe these fibers to be in direct connection with adjacent odontoblasts. If an odontoblast terminates in a sharp point, one offshoot is seen to arise from the point directly connected with a dentinal fiber. If an odontoblast has a broad basis, two or more offshoots may arise from it and run, in the shape of dentinal fibers, into the adjacent dentinal canaliculi. It may also happen that an offshoot of an odontoblast takes another direction, and instead of passing into a dentinal canaliculus, runs parallel with the border of the already formed dentine. Fully developed odontoblasts are not often seen in direct union with dentine, and this is especially true along the lateral portions of the dentinal cap. It is far more common that between the odontoblasts and the dentine medullary corpuscles are present, and the offshoots of the odontoblasts run between these medullary corpuscles in order to reach their respective dentinal canaliculi. Fully developed odontoblasts are rarely seen at the apex of the papilla. Medullary corpuscles alone are usually present in this locality, or they are interposed between the odontoblasts and dentine. Not infrequently we observe layers in the forming dentine, which are represented in Fig. 13.

FIG. 13. *First formed dentine of a human fœtus of six months.*

D. Calcified layer of dentine with marked globular territories.

DT. Dentine composed of a non-calcified basis-substance, likewise globular.

DM. Row of medullary corpuscles before the formation of basis-substance.

O. Odontoblasts, the offshoots of which run between the medullary corpuscles into the dentinal canaliculi.

E. Enamel.

Magnified 1,200 diam.

The odontoblasts have arisen from the fusion of a number of medullary corpuscles, and are present where, at the time, the formation of dentine is not actively in progress. Whenever this is the case, the odontoblasts break up into medullary corpuscles, which are directly transformed into the basis-substance of the dentine. The offshoots of the odontoblasts, which are seen to run directly into the dentinal canaliculi, will appear between the medullary corpuscles as soon as the odontoblasts split up into such corpuscles. The offshoots are formations of living matter seen to emanate from a compact layer at the base and the borders of the odontoblasts. The dentinal fibers remain in situation after the formation of medullary corpuscles, or they are newly formed between the medullary corpuscles, as soon as the dentinal canaliculi are formed. The odontoblasts are not direct dentine formers, but provisional formations, from which arise the medullary corpuscles, and these are changed into the basis-substance of the dentine. Odontoblasts, therefore, the same as ameloblasts, are provisional formations, and dentine, as well as enamel, originates from medullary corpuscles in the same manner in which all forms of connective tissue are known to arise.

It is the rule that the medullary corpuscles are at first transformed into a basis-substance, which is as yet destitute of lime-salts. In specimens stained with carmine, this zone of non-calcified dentine assumes a bright red color, in contradistinction to the calcified portion, which either remains unstained or assumes a greenish tint by the reduction of the chromic acid. Another distinction between the non-calcified and the calcified basis-substance is that the latter has a markedly higher degree of refraction than the former. When we examine such a specimen with high powers of the microscope, we at once become convinced of the identity of the structure of both the non-calcified and the calcified dentine. (See Fig. 5.) We observe a markedly reticular structure in both, and in many instances fine filaments, emanating from the dentinal fibers, are seen to penetrate the reticulum.

At the periphery of the dentine, the bifurcations of the dentinal canaliculi and their tenants are always plainly marked. This feature obviously arises from an aggregation of smaller medullary corpuscles than those appearing at a later period of development. At the same time, the reticulum in the basis-substance is more delicate in the region of the bifurcations, and consequently the meshes of the basis-substance appear narrower in the first-formed dentine than in that which is formed at a later period. We sometimes observe transverse sections of dentine in connection with longitudinal ones, which are of great interest. (See Fig. 14, DC.) Here it is seen that

FIG. 14. *First formed dentine of the molar tooth of a human fœtus of six months.*

DB. Basis-substance of non-calcified dentine.

DL. Basis-substance of calcified dentine, both in longitudinal section.

DC. Calcified dentine in cross section.

Magnified 1,200 diam.

the calcification first starts at the periphery of the fields farthest from the dentinal canaliculi. Thus a comparatively coarse net-work of calcified basis-substance is established, in the meshes of which we observe either an uncalcified basis-substance, or unchanged protoplasm, whereas the central portions of the meshes are occupied by the dentinal fibers in transverse or oblique section. The latter appear larger in inverse ratio to the amount of calcareous matter that has been deposited. From their periphery arise the spokes also seen in fully developed dentine which traverse a light rim—the future canaliculus. The border of the canaliculus is often marked by a circular or crescentic formation, possibly the future rim of elastic substance of the dentinal canaliculi.

The line of the non-calcified basis-substance forming the boundary toward the medullary corpuscles, is found to be either straight, stair-like, or slightly wavy. This feature is never observed close to the periphery of the dentine, but always some distance away from it. These wavy contours unquestionably correspond to the globular territories of which the basis-substance is composed. Previous researches on dissolution of the dentine of temporary teeth, in caries and in the process of eburnitis, especially in the latter, have strongly pointed toward the presence of globular territories in the dentine, the same as in bone tissue. The history of development corroborates the presence of such territories, since they are of very common occurrence in developing dentine of man, as well as of different animals. Their origin is explained in a grouping together of a certain number of medullary corpuscles previous to their transformation into basis-substance. Each globular territory is pierced by a number of dentinal canaliculi, without the least interruption in their course. The writers desire to lay special stress upon the fact that such territories become conspicuous only after calcification of the basis-substance has taken place. Under the theory that the odontoblasts are directly transformed into dentine, the formation of globular territories was inexplicable. In morbid processes, we observe the globular territories of the dentine breaking up into a number of medullary corpuscles. The history of development teaches us that each territory arises from a number of smaller corpuscles. Thus the formation, dissolution, inflammation and reformation of dentine becomes plain.

Since Czermark has drawn attention to uncalcified fields in the dentine of many teeth, and called them "interglobular spaces," a great deal of speculation has been indulged in to explain this occurrence. Such spaces are never present, as far as the writers have observed, close to the periphery of the dentine, but are always found some distance from the bifurcations of the dentinal canaliculi, and sometimes are scattered throughout the dentine. These spaces are filled with a non-calcified basis-substance, or with medullary corpuscles which have not been transformed into basis-substance. The writers often observed within these spaces irregular globular territories, invariably marked by the contours of such territories, but without ever causing a deviation of the course of the dentinal canaliculi.

The origin of such interglobular spaces is traceable to the earliest stages of the formation of the dentine. (See Fig. 15.) In exceptional cases even fully developed dentine will appear composed of layers, or faint concentrically arranged marks in the dentine, traversed without interruption by the dentinal canaliculi. Both the stratification and the interglobular spaces are caused by a faulty deposition of lime-salts in the embryonal development of the dentine. Their cause is probably a temporary interruption of deposition of lime-salts, owing to transient ailments of the mother. These rather anomalous formations of dentine again prove that the basis-substance is made up of globular territories, and these again of medullary corpuscles.

The conclusions which the writers have derived from the studies of the history of the development of dentine, are the following:

I. Dentine from the very issue is a formation of connective tissue, first visible in the shape of a knob-like protuberance termed the papilla.

II. The papilla is composed of medullary tissue, holding an irregular myxomatous net-work, originally scanty, but later on freely supplied with arteries, veins and capillaries.

III. Shortly before the formation of dentine—in the fifth month of fœtal life—there appear at the periphery of the papilla elongated corpuscles, resembling epithelia, which are termed odontoblasts. Where the odontoblasts are in contact with an already formed dentine, they send offshoots into the dentinal canaliculi in varying numbers. These offshoots are dentinal fibers.

IV. The odontoblasts are provisional formations of medullary tissue, which never change directly into dentine, and are therefore visible only where no formation of dentine is going on, or at the time of rest.

V. Before dentine is formed, the odontoblasts break up into medullary corpuscles, and the dentinal fibers, before in connection with the odontoblasts, are located between the medullary corpuscles.

VI. The medullary corpuscles are directly transformed into the basis-substance of the dentine, which at first is destitute of lime-salts, but afterward becomes the seat of a deposition of calcareous matter.

VII. The basis-substance of the dentine is composed of globular territories, the origin of which is traceable in groupings of the medullary corpuscles, shortly before the appearance of the basis-substance.

In the fœtus of the cat, the sheep, and the dog, the process of the formation of dentine is the same as in man, in all essential features. The same rule holds good for the history of the development of dentine in swine. In the latter animals, there is a slight discrepancy as compared with man, mainly in the structure of the papilla. (Fig. 16.)

FIG. 16. *Papilla and adjacent dentine of fœtus of a pig.*

P. Myxomatous tissue of the papilla, with irregularly branching corpuscles, and an abundance of myxomatous basis-substance.

CC. Capillaries, partly fully formed, and partly in the process of formation.

O. Layer of odontoblasts.

D. Dentine.

Magnified 500 diam.

The papilla of a pig fœtus is composed of irregular, branching and partly connecting protoplasmic bodies, somewhat resembling the stellate reticulum of the enamel organ. These corpuscles are uniformly distributed in an abundant mass of basis-substance, which, with lower powers of the microscope, appears finely granular. High powers, however, reveal the delicate network essential to all the varieties of a myxomatous basis substance. This tissue is traversed by a moderate number of blood-vessels, mainly capillaries, which showed in some places stages of development, from the original solid cords of protoplasm to the vacuolization and appearance of endothelia upon their walls.

Toward the periphery of the papilla, spindle-shaped bodies make their appearance, evidently arising (at least to a great extent) from the living matter previously hidden in the myxomatous basis-substance. By an increase in size of one, or the confluence of several such spindle-shaped corpuscles, the odontoblasts arise, and they are often seen in contact with the already formed dentine. Close study of specimens has satisfied the writers that the odontoblasts do not directly form the basis-substance of the dentine, but are merely transitional formations, here, as well as in men, and other animals.

FIG. 17. *Odontoblasts, with adjacent dentine from the fœtus of a pig.*

O. Odontoblasts.

D. Dentine.

Magnified 1,200 diam.

FIG. 18. *Odontoblasts, with adjacent dentine, from the fœtus of a pig.*

O. Odontoblasts.

D. Dentine.

Magnified 1,200 diam.

In the condition of rest, the odontoblasts offer an excellent opportunity to study their relation to the dentine. If they lie against the dentine with a broad basis, four or five offshoots (as represented in Fig. 17) may be seen to enter the adjacent canaliculi, all of which arise from one odontoblast. If, on the contrary, the odontoblasts terminate toward the dentine in a point, a single dentinal fiber will spring therefrom, as represented in Fig. 18. It may also happen that a single odontoblast exhibits no offshoots, in which instance we observe, between the base of the odontoblast and the dentine, delicate filaments which run along the border of the dentine, and from which arise the dentinal fibers. The opposite ends of the odontoblasts terminate in a pointed way, likewise elongated into delicate fibers. These fibers, as well as the lateral portions of the odontoblasts, are inter-connected with all their neighbors by means of delicate thorny offshoots.

## Figures and Tables

**Fig. 12. f1:**
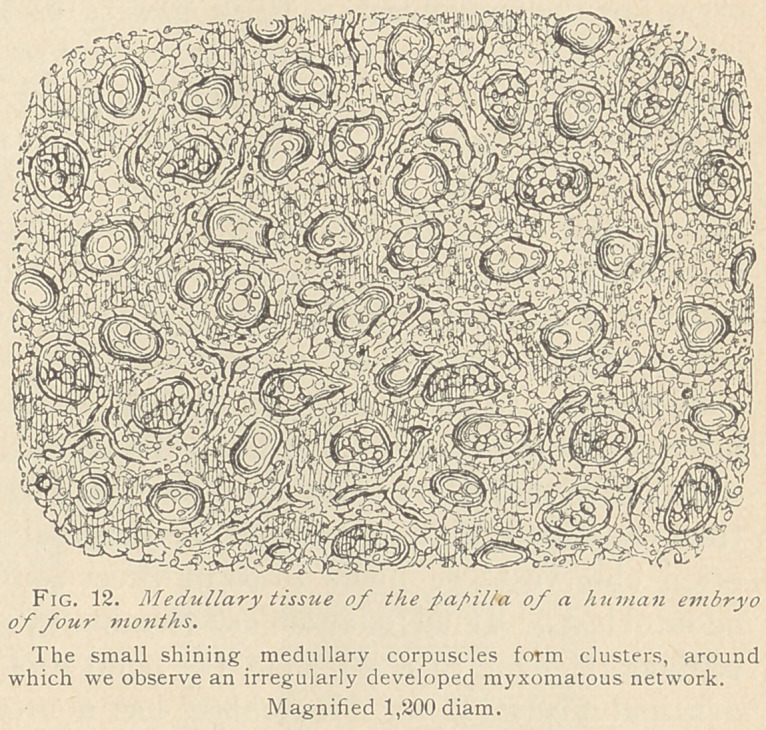


**Fig. 13. f2:**
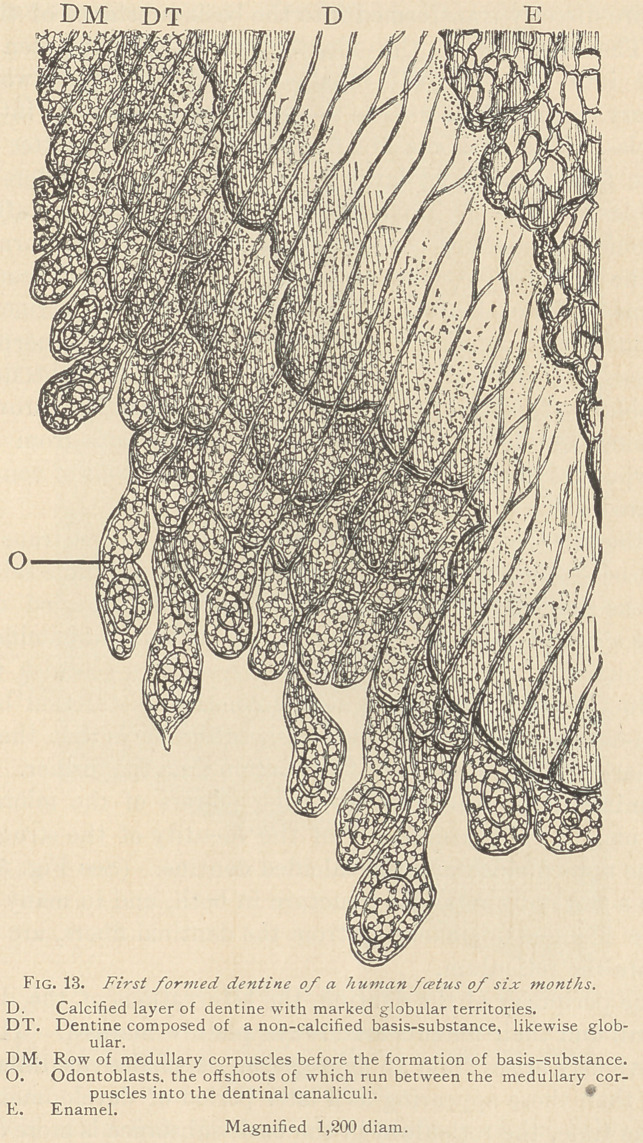


**Fig. 14. f3:**
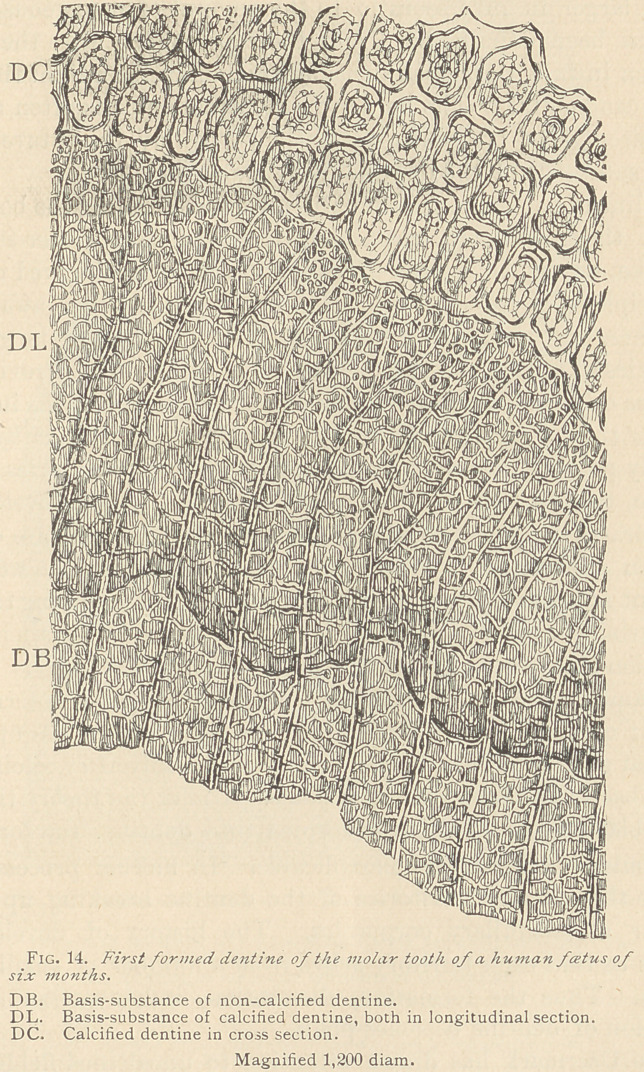


**Fig. 15. f4:**
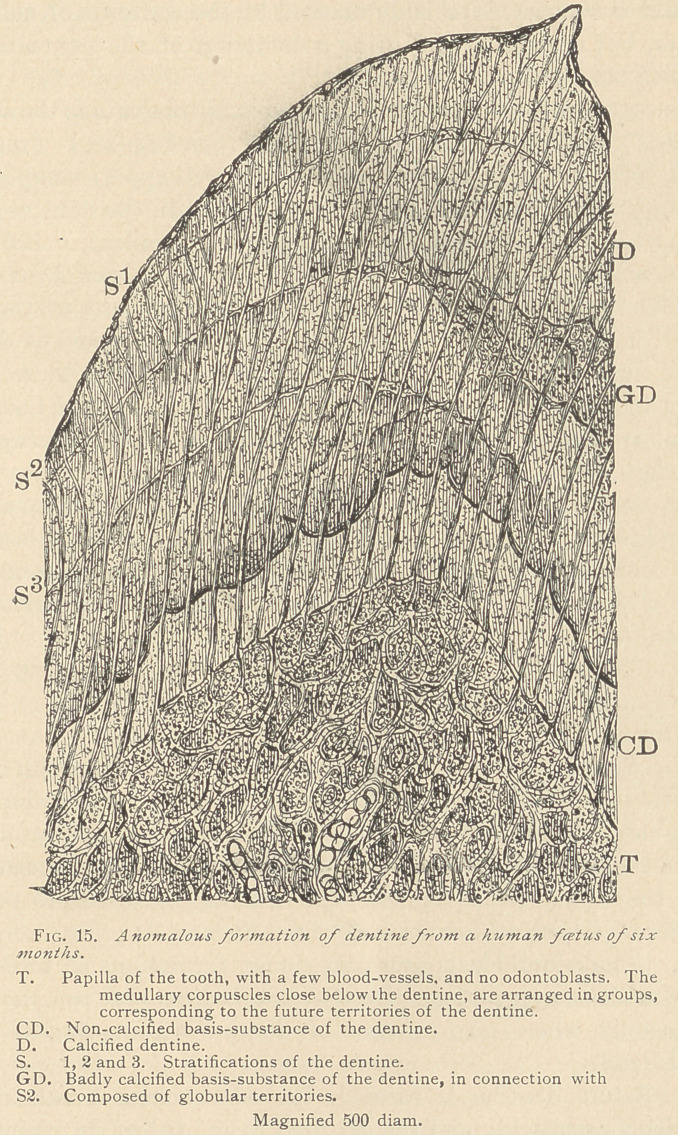


**Fig. 16. f5:**
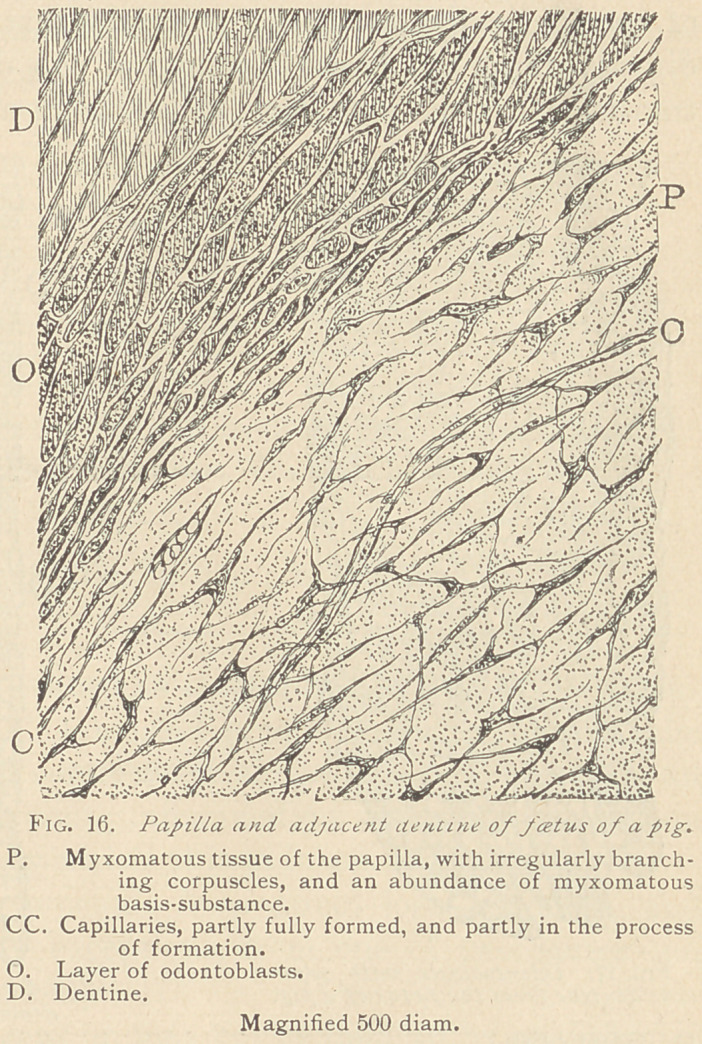


**Fig. 17. f6:**
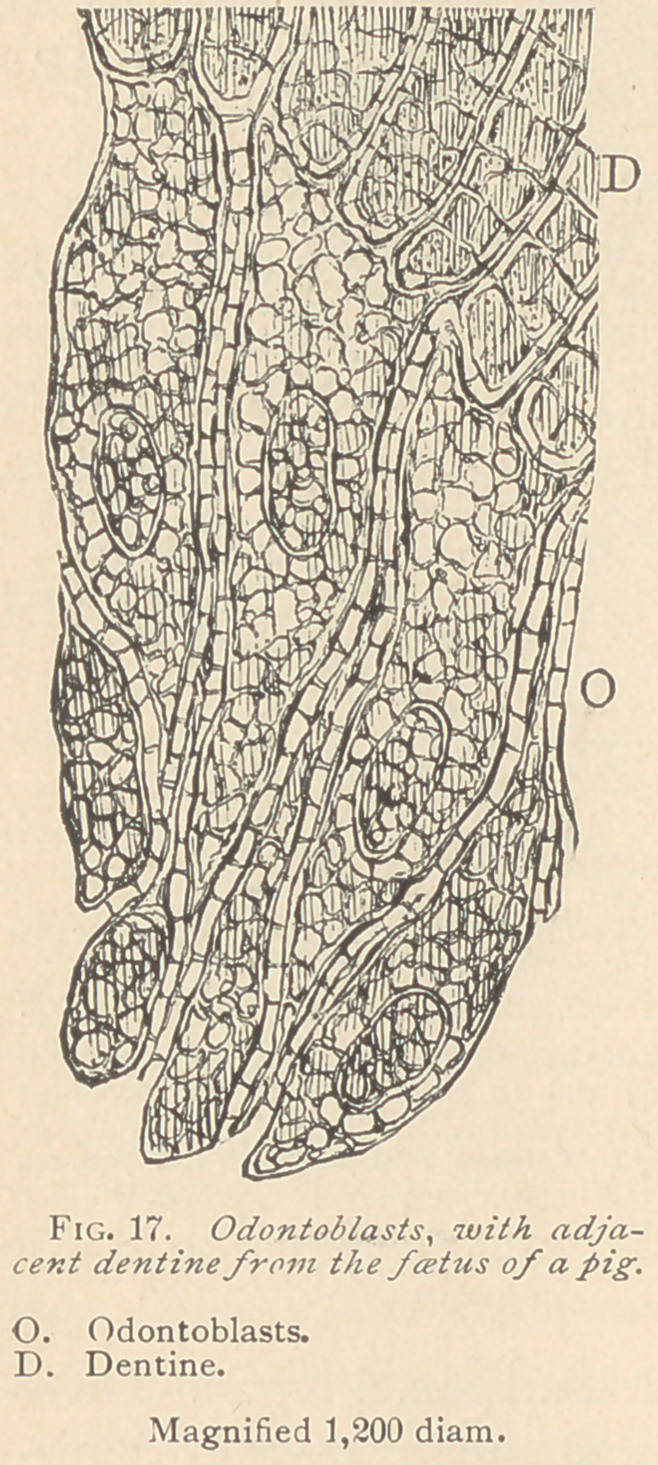


**Fig. 18. f7:**